# Correlations of Differentially Expressed Gap Junction Connexins Cx26, Cx30, Cx32, Cx43 and Cx46 with Breast Cancer Progression and Prognosis

**DOI:** 10.1371/journal.pone.0112541

**Published:** 2014-11-10

**Authors:** Ivett Teleki, Attila Marcell Szasz, Mate Elod Maros, Balazs Gyorffy, Janina Kulka, Nora Meggyeshazi, Gergo Kiszner, Peter Balla, Aliz Samu, Tibor Krenacs

**Affiliations:** 1 1^st^ Department of Pathology & Experimental Cancer Research, Semmelweis University, Budapest, Hungary; 2 2^nd^ Department of Pathology, Semmelweis University, Budapest, Hungary; 3 MTA TTK Lendulet Cancer Biomarker Research Group, Budapest, Hungary; 4 2^nd^ Department of Pediatrics, Semmelweis University, Budapest, Hungary; 5 MTA-SE Pediatrics and Nephrology Research Group, Budapest, Hungary; 6 MTA-SE Tumor Progression Research Group, Budapest, Hungary; Albert Einstein College of Medicine, United States of America

## Abstract

**Background and Aims:**

Connexins and their cell membrane channels contribute to the control of cell proliferation and compartmental functions in breast glands and their deregulation is linked to breast carcinogenesis. Our aim was to correlate connexin expression with tumor progression and prognosis in primary breast cancers.

**Materials and Methods:**

Meta-analysis of connexin isotype expression data of 1809 and 1899 breast cancers from the Affymetrix and Illumina array platforms, respectively, was performed. Expressed connexins were also monitored at the protein level in tissue microarrays of 127 patients equally representing all tumor grades, using immunofluorescence and multilayer, multichannel digital microscopy. Prognostic correlations were plotted in Kaplan-Meier curves and tested using the log-rank test and cox-regression analysis in univariate and multivariate models.

**Results:**

The expression of GJA1/Cx43, GJA3/Cx46 and GJB2/Cx26 and, for the first time, GJA6/Cx30 and GJB1/Cx32 was revealed both in normal human mammary glands and breast carcinomas. Within their subfamilies these connexins can form homo- and heterocellular epithelial channels. In cancer, the array datasets cross-validated each other’s prognostic results. In line with the significant correlations found at mRNA level, elevated Cx43 protein levels were linked with significantly improved breast cancer outcome, offering Cx43 protein detection as an independent prognostic marker stronger than vascular invasion or necrosis. As a contrary, elevated Cx30 mRNA and protein levels were associated with a reduced disease outcome offering Cx30 protein detection as an independent prognostic marker outperforming mitotic index and necrosis. Elevated versus low Cx43 protein levels allowed the stratification of grade 2 tumors into good and poor relapse free survival subgroups, respectively. Also, elevated versus low Cx30 levels stratified grade 3 patients into poor and good overall survival subgroups, respectively.

**Conclusion:**

Differential expression of Cx43 and Cx30 may serve as potential positive and negative prognostic markers, respectively, for a clinically relevant stratification of breast cancers.

## Introduction

Breast cancer is one of the most fatal malignancies of women in the economically developed countries [1]. In addition to clinicopathological factors, molecular techniques allow clinically relevant subtyping of breast cancers by testing for biomarkers of tumor prognosis and response to therapy (prediction) [2], [3]. However, despite accurate testing, only ∼50% of the selected cases respond e.g. to anti-Her2 immunotherapy [4]. Therefore, further stratification within breast cancer subtypes is needed to assist in selecting more personalized treatment options and revealing the background of therapy resistance.

Homeostasis in breast tissue requires regulated direct cell-cell interactions. Abnormal expression of adherent (E-cadherin) and tight junction proteins (claudins) in mammary glands has been demonstrated to contribute to breast cancer development and to assist in clinical subtyping [5], [6]. Recently, we have found that monitoring connexin (Cx) proteins Cx26 and Cx46 in neoadjuvant treated breast cancer allowed refinement of intermediate prognostic subgroups of residual tumor classifications [7]. Connexins and their cell membrane channels play essential roles in the control of cell proliferation, differentiation and apoptosis and their deregulation can contribute to carcinogenesis including breast cancers [8], [9]. However, no comprehensive study correlating connexin mRNA and protein levels with breast cancer progression and prognosis have been published.

Six of the tetraspan transmembrane connexins form hemichannels which can align for gap junctions in adjacent cells allowing the orderly transport of ∼<1,8 kDa regulatory molecules between coupled cells including ions, metabolites (nucleotides, linear oligopeptides), second messengers (c-AMP, IP_3_ and Ca^2+^) and morphogenes [10], [11]. Connexins may also function as hemichannels or through intracellular protein-protein interactions with oncogene products such as Src, signaling protein kinases and cytoskeletal elements [12]–[14]. More than one of the 21 cloned connexin isotypes are expressed in most human cell types [15] and their importance is reflected by their ubiquitous presence and large density in all solid tissues, early emergence during embryogenesis and high evolutionary conservation throughout vertebrates [10]. Principal connexin functions are related to the maintenance of cell homeostasis and integration of compartmental activities within cell networks [16].

Connexins and gap junctions have long been implicated in tumor suppression [17]. Though connexins can be upregulated in dysplasia or early cancer [18], their expression and functions are usually reduced in malignant tumors [19] and can be aborted in advanced cancers [20]. However, recent observations suggest a context dependent regulation of connexins in cancer with occasional stage dependent up-regulation [13]. Furthermore, connexin isotypes not found in the normal tissue may also emerge in the related cancer [21].

Available data on connexin expression in normal breast and breast cancer are controversial. Limitations of large scale screening of connexins are explained by scarce antibodies detecting their isotypes in archived tissues and difficulties of resolving the small (<1 µm) connexin plaques in ∼5 µm thick sections. So far, Cx43 and Cx26 have been detected to contribute to human [22]–[25] and Cx30 and Cx32 to mouse mammary gland development and lactation [26]. In primary breast cancers Cx43 and Cx26 have been suggested as tumor suppressors [27], [28]. However, increased Cx43, Cx26 and Cx32 protein levels have also been found in lymph node metastases compared to primary breast cancers [29], [30] but without correlation to disease prognosis [8]. Recently, Cx46 has also been implicated in the adaptation of breast cancer cells to hypoxia [31]. Furthermore, heterocellular communication between breast carcinoma cells and vascular endothelia has been confirmed during metastatic tumor invasion [32], [33].

Here, we tested publically available mRNA expression array databases and tissue microarray (TMA) series of breast cancers for connexin isotype expression. Based on mRNA expression data, a comprehensive screening for five connexin isotypes, GJA1/Cx43, GJA3/Cx46, GJB2/Cx26, GJA6/Cx30 and GJB1/Cx32 was performed at the protein level in normal pre-menopausal breast glands and in a cohort of cancers representing all grades and major breast cancer subtypes. Differential connexin expression showed significant correlations with tumor progression and disease outcome for potential utilization in breast cancer diagnostics and treatment design.

## Materials and Methods

### *In silico* connexin mRNA expression data analysis in breast cancer

An *in silico* analysis was carried out using the web-based Kaplan-Meier Plotter (http://kmplot.com) [34] utilizing publicly available gene expression data of HGU133A and HGU133+2 microarrays (Affymetrix, Santa Clara, CA) and survival information of 1809 patients from gene expression omnibus (GEO) (**Table 1**). Expression data were available for Cx32, Cx43 and Cx46. High and low expression groups were separated along the median connexin expression for each isotype (**Fig. S1**) and investigated in context with relapse free survival (RFS), overall survival (OS) and distant metastasis free survival (DMFS). Data for Cx26 and Cx30 mRNA expression of 1899 breast cancers, published in the METABRIC project using the Illumina HT-12 v3 platform (San Diego, CA) were downloaded from the European Genome-Phenome Archive [35]. Overlapping data on Cx32, Cx43 and Cx46 were used to verify the Affymetrix array results. Expression data were filtered by ER and HER2 status, major subtypes, lymph node involvement and/or tumor grade. Patient datasets were also grouped according to systematically untreated cases, endocrine-treated ER positive cases and a patient cohort similar to SEER (Surveillance Epidemiology and End Results) prevalence. The clinicopathological features of tumor samples tested for connexin mRNA expression are summarized in **Table 1**.

**Table 1:** Primary breast cancers tested for connexin expression using *in silico* microarray datasets for mRNA or tissue microarray sections for protein analysis.

	*In silico* datasets		
Cohort	Affymetrix	Illumina	TMA samples
Patients (n)	1809	1988	127
Age (years ± SD)	57±13	61.8 (median)	59±12
Follow-up time (months ± SD)	123±82	86.4 (median)	101±40
Relapse/Death event (n %)	690 (38.1%)	643 (32.3%)	42 (33.1%)
Grade 1	198 (10.9%)	170 (8.6%)	41 (32.3%)
Grade 2	534 (29.5%)	775 (39%)	41 (32.3%)
Grade 3	312 (17.2%)	954 (48%)	44 (34.6%)
Data not provided	745 (41.2%)	89 (4,4%)	1 (0.8%)
IDC	0	0	88 (69.3%)
ILC	0	0	13 (10.2%)
Other	0	0	14 (11.0%)
Data not provided	1809	1988	12 (9.4%)
ER positive	968 (53.5%)	1517 (76.3%)	89 (70.1%)
Luminal A	969 (53.6%)	825 (41.5%)	70 (56%)
Luminal B	536 (29.6%)	668 (33.6%)	19 (15.2%)
ER negative	578 (31.9%)	471 (23.7%)	36 (28∶3%)
HER2 positive	295 (16.3%)	242 (12.1%)	15 (12%)
TNBC	230 (12.7%)	331 (16.7%)	21 (16.8%)
Unknown	0	0	2 (1.6%)

TMA: tissue microarray; SD: standard deviation; IDC: invasive ductal carcinoma.

ILC: invasive lobular carcinoma; ER: estrogen receptor; TNBC: triple negative basal cell type.

Note: Technically unsatisfactory samples were left out.

### Breast cancer samples tested for protein expression

This study was approved (#85/2007) by the Institutional Review Board (IKEB) of Semmelweis University (Budapest, Hungary). The Regional Committee of Science and Research Ethics waived the need for individual patient consent on archived tissues available for primary diagnostics for further testing of potential biomarkers.

Formalin-fixed, paraffin-embedded (FFPE) tissue samples of 127 primary breast cancers collected between 1999 and 2002 at Buda MAV Hospital, Budapest, Hungary were studied (**Table 1**). This study was approved (#85/2007) by the Institutional Review Board (IKEB) of Semmelweis University (Budapest, Hungary). Patients were subjected to partial or total mastectomy optionally accompanied by axillary block dissection. Fifty patients (39.4%) underwent operation without any supplementary treatment, 16 patients (12.6%) received irradiation only, 12 patients (9.4%) received taxane- or antracycline-based chemotherapy only, 40 patients (31.5%) were subjected to trimodality treatment and in 9 patients (7.1%) there was no treatment information available. Breast carcinomas were immunophenotypically classified into 4 subgroups. Luminal A phenotype (estrogen/ER -and progesterone/PR receptor positive, epidermal growth factor receptor 2/HER2 negative tumors with Ki67 expression in <20% of tumor cells) was detected in 70 cases, luminal B subtype (ER/PR and HER2 double positive tumors; or ER/PR positive and HER2 negative tumors with >20% Ki67 positive tumor cells) was established in 19 cases, triple negative subtype (ER/PR negative and FISH confirmed HER2 negative tumors) was found in 21 cases and HER2 positive subtype (ER and PR negative and immunohistochemically HER2 3+ or 2+ cases where gene amplification was confirmed by FISH) was detected in 15 cases. Immunophenotype was not available for two patients. At least duplicate cores of 2 mm diameter were collected from the archived tissue blocks into TMAs using a manual array builder (Histopathology Ltd., Pecs, Hungary) including 41 grade1, 41 grade2 and 44 grade3 tumors. Normal mammary glands of 3 pre-menopausal women were also examined for connexin expression. The clinicopathological features of tumor samples tested are shown in **Table 1**.

### Immunofluorescence detection of connexins and cell proliferation

To resolve the small size (frequently <1 µm) connexin plaques through the 4 µm thick sections in a large number of samples without fading a sensitive immunofluorescence detection was set up combined with multilayer (3–5 layers) whole slide digitalization for permanent archives using Pannoramic Scan (3DHISTECH).

TMA slides were routinely de-waxed in xylene and rehydrated through graded ethanol series. Antigen unmasking was done in a buffer containing 0.1 M Tris and 0.01 M EDTA (pH 9.0) using an electric pressure cooker (Avair, Biofa, Veszprem, Hungary) for 20 min at ∼105°C. Slides were then briefly digested using 0.25% Gibco trypsin phenol red (1∶50, Life Technologies, Carlsbad, CA, Ref: 25050-014) for 10 sec. After protein blocking in 1% BSA-TBS (0.1 M Tris-buffered saline, pH7.4) for 20 min, the slides were incubated overnight with antibodies, validated for isotype size in western blots, recognizing human or rat connexin sequences including mouse anti-Cx26 (1∶500, clone: CX-1E8, Invitrogen/−Life Technologies, Eugene, OR), and rabbit anti-Cx30 (1∶75, code: HPA014846, Sigma-Aldrich, St Luis, MO), -Cx32 (1∶30, code: HPA010663, Sigma-Aldrich), -Cx43 (1∶100, code: #3512, Cell Signaling, Beverly, MA) and -Cx46 (1∶100, code: SAB1300557, Sigma-Aldrich). Mammal connexin-specific antibodies show high degree of cross-reactivity with the relevant human connexins [15]. The proliferation marker Ki67 protein was also simultaneously detected using the mouse anti-human Mib1 clone (1∶2 ready-to-use, code: IR626, Dako, Glostrup, Denmark), or the rabbit SP6 clone (Thermo-LabVision, Fremount, CA) combined with the mouse anti-Cx26 antibody. For double antigen detection slides were incubated with a mixture of Alexa Fluor 564 goat anti-rabbit IgG (red, code: A11035) and Alexa Fluor 488 goat anti-mouse IgG (green, code: A11001) diluted in 1∶200, for 90 min. Cell nuclei were stained using Hoescht (blue, code: B2883) in 1∶1000 for 90 sec (all fluorochrome labeled reagents were from Invitrogen-life Technologies). All incubations were done in humidity chambers at room temperature and slides were washed between the steps using 0.1 M TBS (Tris-buffered saline) pH 7.4 for 2×5 min.

### Evaluation of connexins and cell proliferation in breast cancer

Connexins are known to form transmembrane hemichannels and gap junctions and can directly interact with regulatory proteins within cells resulting in particulate cell membrane and cytoplasmic signals [13]. Both localizations were taken into account at analysis. Connexin expression was tested in digital slides using Pannoramic Viewer (version 1.15) software on a 4-scale scoring system considering the frequency of positive tumor cells. Score 0: <5% positive cells; +1∶5–20% positive cells; +2∶21–50% positive cells; +3: >50%.

The Ki67 immunoreaction was evaluated using a linear 10-scale scoring system based also on the positive tumor cell fractions (Score 0∶0, 1∶0–1%, 2∶1–5%, 3∶6–10%, 4∶11–15%, 5∶16–20%, 6∶21–33%, 7∶34–50%, 8∶51–66%, 9∶67–80%, 10∶81–100%).

### Statistical analysis

The gene chip mRNA expression results were analyzed within the R statistical environment (R version 2.10.1; R Foundation for Statistical Computing, Vienna, Austria) as described previously [36]. Results were shown in Kaplan-Meier plots including the hazard ratios and P of the log-rank test. For statistical testing of protein expression scores gained in TMA sections of breast cancer samples the SPSS 15.0 software was used (SPSS Inc., Chicago, IL). Connexin isotype expression was correlated both with protein levels in normal mammary glands and with clinicopathological features including grade, hormone receptor status (HR), presence of necrosis and invasion, Nottingham Prognostic index (NPI) and mitotic index (MI) using the Spearman-rank test. Prognostic relevance of connexin isotype expression estimating overall survival (OS), disease-free survival (DFS) and distant metastasis free survival (DMFS) were evaluated with the log-rank test. Cox proportional hazard regression was used to quantify the influence of variables in univariate analysis and to assess those in multivariate models which were significantly associated with survival but without direct significant pairwise correlations when using the Spearman’s rank test. Hazard ratios (HR) were given with 95% confidence intervals (95% CI). P values of <0.05 were considered statistically significant in all tests.

## Results

### *In silico* analysis of connexin mRNA expression in breast cancer

#### Affymetrix dataset

**Figure 1:**
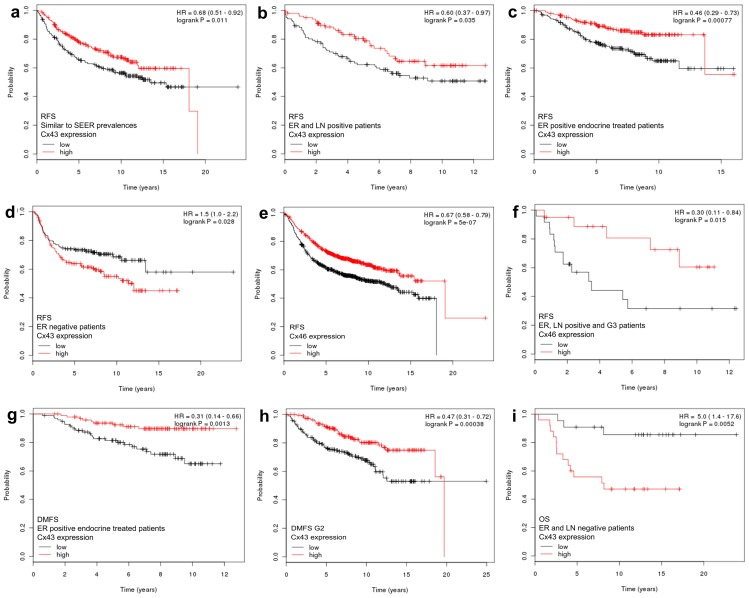
Kaplan-Meier plots of significant prognostic correlations of elevated (>median) Cx43 (a-d and g–i) and Cx46 (e–f) mRNA expression in 1809 breast cancers based on *in silico* analysis of Affymetrix array data. Cx43 expression was associated with improved relapse/disease free survival (RFS) in a tumor group similar to SEER prevalence (a), in ER and lymph node positive tumors (b) and in ER positive endocrine treated tumors (c), but reduced RFS in ER negative tumors (d). Elevated Cx46 levels were predictive for better RFS in the whole patient cohort (e) and in ER and lymph node positive grade 3 patients (f). Elevated Cx43 mRNA levels were also correlated with significantly better distant metastasis-free survival (DMFS) in ER positive endocrine treated patients (g) and in grade 2 cancers (h). Cx43 expression in ER negative patients was linked with reduced overall survival (OS) (i). Significance (at p<0.05) was calculated using the log-rank test. HR: hazard ratio (at 95% Confidence Interval).

Correlates between connexin mRNA expression and disease prognosis are summarized in **Table 2** and some are highlighted in Kaplan-Meier plots. Significantly better RFS was linked with tumors showing: elevated (>median) Cx32 mRNA levels in the whole patient cohort; elevated Cx43 mRNA levels in a tumor group similar to SEER prevalence (**Fig. 1a**), in the ER positive tumors, in the Luminal A group, in the ER and lymph node positive tumors (**Fig. 1b**) and in ER positive endocrine treated tumors (**Fig. 1c**). Elevated Cx43 mRNA expression was associated with shorter RFS only in the ER negative group (**Fig. 1d**). Increased Cx46 expression was also predictive for longer RFS in the whole patient cohort (**Fig. 1e**) and in the ER and lymph node double positive grade 3 patients (**Fig. 1f**). Elevated Cx43 mRNA levels were also associated with significantly longer DMFS in the whole patient cohort, in the lymph node negative patients, in the ER positive endocrine-treated patients (**Fig. 1** **g**) and in grade 2 cancers (**Fig. 1** **h**). Again, in ER negative patients Cx43 expression showed an inverse correlation with prognosis (OS) (**Fig. 1i**).

**Table 2:** Significant prognostic correlations of connexin mRNA expression data resulting from the *in silico* analysis of 1809 (Affymetrix) and 1988 (Illumina) breast cancers.

Connexin isotype	Significant prognostic correlations		Hazard ratio	95% Confidence	Log-rank P
	Positive	Negative		interval	(significance)
GJA1 (Cx43)	All patients (I)		0.67	0.57–0.79	2.5e-06
	All patients (A/M)		0.63	0.47–0.85	0.002
	SEER prevalence (A)		0.68	0.51–0.92	0.011
	ER positive (A)		0.79	0.63–0.99	0.036
	ER positive (I)		0.68	0.56–0.83	9.3e-05
	Luminal A (I)		0.70	0.52–0.93	0.015
	G2 tumors (A/M)		0.47	0.31–0.72	0.00038
	ER & LN positive (A)		0.63	0.41–0.98	0.039
	Endocrine treated (A)		0.46	0.29–0.73	0.00077
	Endocrine treated (A/M)		0.31	0.14–0.66	0.0013
	Endocrine treated (I)		0.63	0.49–0.84	0.0004
		ER negative (A)	1.5	1.00–2.20	0.028
		ER & LN negative (A/OS)	5.0	1.40–17.6	0.0052
		ER negative (I)	1.31	0.96–1.18	0.09 (trend)
GJA3 (Cx46)	All patients (A)		0.67	0.58–0.79	5,00E-07
	All patients (I)		0.83	0.70–0.97	0.021
	Luminal A (I)		0.72	0.53–0.98	0.036
	ER negative (I)		0.74	0.55–0.99	0.045
	HER2 positive (I)		0.66	0.45–0.95	0.026
	Chemotherapy (I)		0.73	0.55–0.98	0.035
GJB1 (Cx32)	All patients (A)		0.63	0.54–0.73	2.4e-09
	All patients (I)		0.81	0.70–0.95	0.0095
	ER positive (I)		0.82	0.68–0.99	0.043
	Luminal B (I)		0.77	0.60–0.98	0.034
	Endocrine treated (I)		0.81	0.66–1.00	0.046
GJB2 (CX26)		Luminal B (I)	1.40	1.10–1.80	0.012
		All patients (I)	1.20	1.00–1.40	0.058 (trend)
GJB6 (Cx30)		All patients (I)	1.20	1.10–1.50	0.0088
		ER positive (I)	1.40	1.10–1.70	0.0012
		Luminal A (I)	1.50	1.10–2.10	0.0088
		Luminal B (I)	1.50	1.10–1.90	0.0058
		Endocrine treated	1.40	1.20–1.80	0.00082
	HER2 positive		0.73	0.50–1.06	0.1 (trend)
	Triple negative		0.72	0.49–1.05	0.085 (trend)

A: Affymetrix platform using relapse-free survival (RFS) data if not indicated, distant metastasis-free survival (DMFS) data indicated as M or overall survival data (OS).

I: Illumina platform using OS data only (not indicated).

#### Illumina dataset

**Figure 2:**
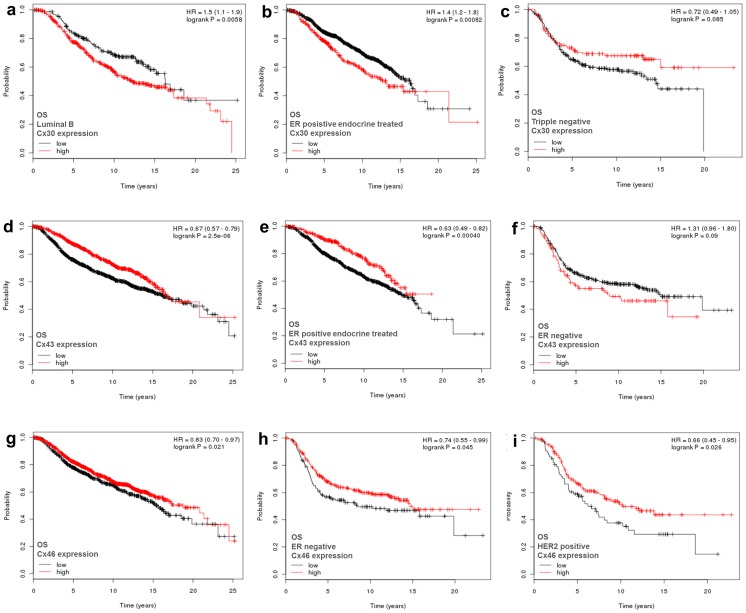
Kaplan-Meier plots of significant correlations between overall survival (OS) and elevated (>median) Cx30 (a–c), Cx43 (d–f) and Cx46 (g–i) mRNA expression in 1988 breast cancers based on *in silico* analysis of Illumina array data. Elevated Cx30 levels were linked with reduced OS in patients with luminal B tumors (a) and in ER positive endocrine therapy treated patients (b), but with a strong tendency for improved OS in ER negative cases (c). Cx43 expression was associated with better OS in the whole cohort (d) and in ER positive endocrine treated patients (e) but showed a strong trend for reduced OS in ER negative patients (f). Cx46 expression was associated with improved OS in the whole cohort (g), in ER negative patients (h) and in HER2 positive patients (i). Significance (at p<0.05) was calculated using the log-rank test. HR: hazard ratio (at 95% Confidence Interval).

Significant prognostic correlations of Cx26 and Cx30 mRNA expression, data not available in the Affymetrix set, and of Cx32, Cx43 and Cx46 are summarized in **Table 2**. Except for a significant inverse correlation with OS in luminal B tumors, Cx26 mRNA expression did not show significant link with breast cancer prognosis. Cx30 mRNA levels, however, were inversely correlated with OS in the whole cohort, in the ER positive patients, both in the Luminal A and Luminal B patient subgroups (**Fig. 2a**) and in the ER positive endocrine therapy treated patients (**Fig. 2b**). As a contrary, elevated Cx30 mRNA levels were associated with a strong tendency of longer OS in the ER negative group including the HER2 positive and the triple negative (**Fig. 1c**) patient groups.

Prognostic OS data of the Illumina platform on Cx43, Cx46 and Cx32 expression correlated well with DFS figures of the Affymetrix platform. Accordingly, elevated Cx43 levels showed a significant positive association with OS in the whole patient cohort (**Fig. 2d**), in the ER positive group, in the luminal A tumors and in the ER positive endocrine therapy treated patients (**Fig.** **2e**). However, elevated Cx43 levels showed an inverse statistical trend with OS in the ER negative cases (**Fig. 2f**) and in the triple negative cases. Elevated Cx46 expression was associated with improved OS in the whole patient cohort (**Fig. 2** **g**), in the Luminal A group, in the ER negative patients (**Fig. 2** **h**), in the HER2 positive patients (**Fig. 2i**) and in the chemotherapy treated patients. Increased Cx32 expression was also linked with favorable OS in the whole cohort, in the ER positive patients, mainly in the Luminal B subgroup and in the ER positive endocrine therapy treated patients.

### Expression of connexin isoproteins in normal mammary gland

**Figure 3:**
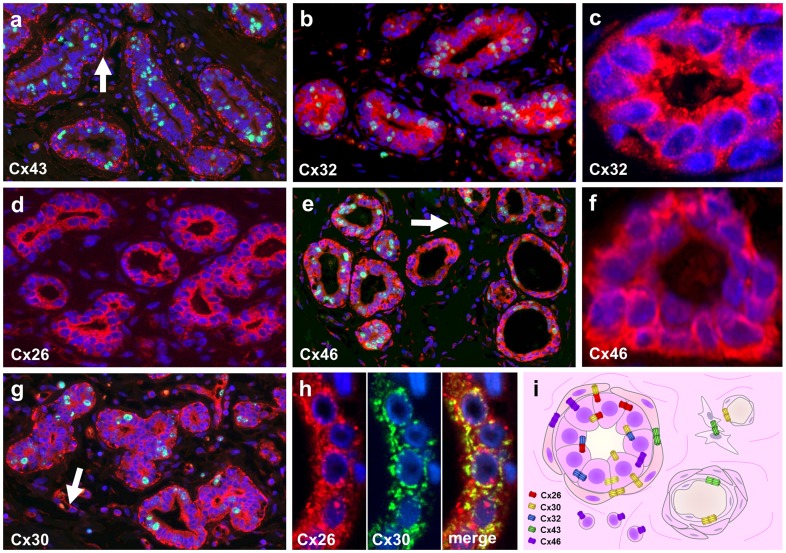
Detection of connexin protein isotypes (Alexa-564, red) and the proliferation marker Ki67 protein (Alexa-518, green) in normal pre-menopausal breast tissue. Punctuate Cx43 reaction was localized to the myoepithelial cell layer of normal mammary glands and to adjacent stromal cells (arrow) (a). Both Cx32 (b and c) and Cx26 (d) proteins were found dominantly in the luminal epithelial cells. Cx46 protein was found both in the myoepithelial and luminal cells and less in stromal inflammatory cells (arrow) (e and f). High power views show Cx32 mainly linked to the luminal cells (c) and Cx46 mainly localized to the basal cells (f) involving both their cytoplasm and intercellular borders. Cx30 was revealed along myoepithelial cells and at the apex of luminal epithelium and less in the rest of luminal cells, and along vascular endothelial cells (arrow) (g). Co-localization of Cx26 (red) and Cx30 (green) in epithelial cells (yellow), involving the intercellular borders (h). Double immunofluorescence, cell nuclei are stained blue using Hoescht. Summary drawing of our results shows potential homo- or heterocellular/typic interactions of Cx26 (red), Cx32 (blue), Cx46 (violet), Cx43 (green) and Cx30 (yellow) gap junctions in a normal mammary gland (i).

In the normal mammary gland connexin proteins were primarily localized to the cell membranes using immunofluorescence. We confirmed Cx43 expression along the myoepithelial cell layer and between stromal and endothelial cells (**Fig. 3a**), and Cx26 expression between the luminal epithelial cells (**Fig. 3b**). In addition, we detected, for the first time, Cx32 protein between the luminal epithelial cells (**Fig. 3c**), Cx30 in the myoepithelial cells and some in the luminal cells concentrating at their apex (**Fig. 3d**) and Cx46 protein both in the myoepithelial and luminal epithelial cells and in some stromal inflammatory cells (**Fig. 3e**). These findings are summarized in a drawing in **Fig. 3f**.

### Prognostic and clinicopathological correlates of connexin expression in breast cancer

**Figure 4:**
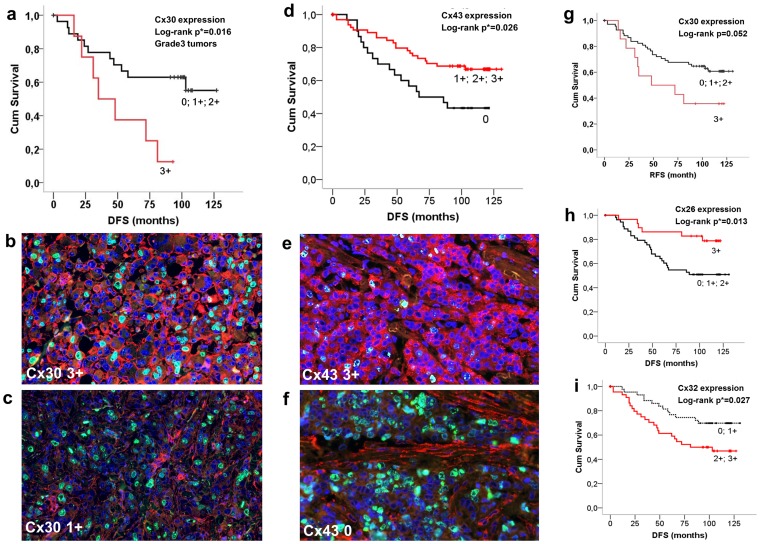
Detection of connexin isoproteins in invasive breast cancers (b, c, e and f) and their significant correlations with relapse/disease free survival (RFS) shown in Kaplan-Meier plots (a, d and g–i). Elevated Cx30 expression (score 3+) was linked with reduced RFS in grade 3 patients (a–c) and with a strong positive trend in the whole cohort (g). Strong cell membrane and less cytoplasmic Cx43 staining (Alexa 564, red) in b (3+) compared to c (1+) in highly proliferating (Ki67– Alexa 518, green) grade3 tumors. Cx43 expression (scores 1–3+) was associated with improved RFS in the whole cohort (d–f). Strong cytoplasmic and less cell membrane Cx43 staining (red) in a tumor with low proliferation rate (green nuclei) (e) and stromal Cx43 signal only in a highly proliferating tumor (f ). Unlike at mRNA level, elevated Cx26 signal (score 3+) correlated with improved RFS (h), while medium to high Cx32 levels (scores 2–3+) showed a negative link with RFS in the whole patient cohort (i). Significance (at p<0.05) was calculated using the log-rank test. Double immunofluorescence, cell nuclei are labeled blue using Hoescht.

Connexin proteins were localized either at the cell membrane or in the cytoplasm or both in breast carcinoma cells. Log-rank test was used for revealing their prognostic associations and Spearman-rank test (ρ) for their correlations with clinicopathological variables. In line with mRNA data, there was a significant statistical link between Cx30 expression (score 3+) and reduced RFS in grade 3 patients (p = 0.016) (**Fig. 4a–c**) and a strong trend concerning the whole patient cohort (p = 0.052) (**Fig. 4** **g**). High Cx30 levels (score 3+) also demonstrated a positive correlation with mitotic index (MI) (*ρ = *0.29). Likewise, confirming mRNA data, Cx43 expression (scores 1–3+) was associated with significantly longer RFS in the whole patient cohort (p = 0.026) (**Fig. 4d–f**) and in grade 2 tumors (p = 0.032); and it correlated positively with HR levels (ρ = 0.23) and negatively with tumor grade (ρ = −0.22). Cx46 expression displayed a negative correlation with tumor grade (ρ = −0.2) but only a non-significant positive trend with disease prognosis.

Discordant prognostic results were found between mRNA and protein levels concerning the Cx26 and Cx32 isotypes. High Cx26 levels (score 3+) correlated with significantly improved RFS in the whole patient cohort (p = 0.013) (**Fig. 4** **h**), in the ER positive (p = 0.007) and in Luminal A (p = 0.017) tumors (Fig. 4a–c). Also, there was a negative correlation between Cx26 expression and NPI (ρ = −0.25). Medium to high Cx32 levels (scores 2–3+) showed a significant negative correlation with RFS in the whole cohort (p = 0.033) (**Fig. 4i**) and in Lumina B-Her2 positive tumors (p = 0.025); and correlated positively with tumor grade (*ρ = *0.34), the presence of necrosis (*ρ* = 0.19), NPI (*ρ* = 0.28), mitotic index (*ρ* = 0.27) and Ki67 expression (*ρ* = 0.21) and negatively with hormone receptor levels (*ρ* = −0.3).

### Prognostic power of connexins in relation to current prognostic factors

Traditionally tested factors such as NPI value of >4, MI of >25 and Ki67 expression in >50% tumor cells (score 7 in our evaluation) were statistically linked with worse RFS (p_NPI_ = 0.011, p_MI = _0.002, p_Ki67_ = 0.001), while hormone receptor positivity was linked to longer RFS (p = 0.002).

Results of Cox multivariate analysis in relation to RFS are summarized in **Table 3**. Cx26, Cx30 and Cx43 expression could be examined against necrosis, vascular invasion, MI, Ki67 index and HR status, and Cx32 against vascular invasion. In breast cancers, >50% Cx26 positive tumor cells (score 3) or >5% Cx43 positive tumor cells (scores 1–3) proved to be significantly stronger independent prognostic factors, than vascular invasion or necrosis. Furthermore, score 3 Cx26 positivity in tumor cells was found nearly as a strong prognostic factor as HR or Ki67 index. Expression of Cx43 in >50% of tumor cells (score 3) was also confirmed nearly as a strong prognostic factor as MI. Score 3+ (>50%) Cx30 positivity in grade 3 tumors proved to be a stronger independent negative prognostic factor than MI or necrosis. Furthermore, Cx32 positivity in <20% Cx32 positive tumor cells (scores 0–1) was confirmed to be a stronger prognostic factor than vascular invasion.

**Table 3:** Prognostic value of connexin protein isotype expression using multivariate Cox regression analysis.

Parameter	p-value	HR	95% CI	
			Lower	Upper
Cx43 (0_123)	0.027	0.470	0.241	0.916
Vascular invasion	0.371	0.718	0.348	1.483
Necrosis	0.643	1.184	0.579	2.419
Cx43 (012_3)	0.048	0.518	0.271	0.993
Vascular invasion	0.474	0.766	0.369	1.589
Mitotic index	0.329	1.386	0.719	2.671
Cx26 (012_3)	0.022	0.354	0.146	0.863
Vascular invasion	0.047	0.404	0.165	0.987
Hormone receptor	0.007	0.368	0.179	0.759
Cx26 (012_3)	0.015	0.329	0.134	0.809
Vascular invasion	0.073	0.443	0.182	1.079
Necrosis	0.395	1.402	0.643	3.058
Ki67	0.009	5.384	1.535	18.881
Cx26 (012_3)	0.019	0.314	0.119	0.829
Vascular invasion	0.091	0.462	0.189	1.130
Cx30 (012_3)	0.016	4.209	1.309	13.539
Mitotic index	0.885	0.856	0.103	7.102
Necrosis	0.178	2.153	0.706	6.569
Cx32 (01_23)	0.032	2.103	1.064	4.155
Vascular invasion	0.319	0.670	0.305	1.471

HR: hazard ratio; CI: confidence interval;

0123: score categories where _ represents thresholds.

## Discussion

Despite testing for traditional and molecular prognostic and predictive markers, the heterogeneity and therapy resistance urges for further molecular stratification of breast cancers [37], [38]. Deregulation of connexins and their cell membrane channels has been implicated in breast carcinogenesis and tumor progression, however, prognostic correlations of connexins have been rarely found [9], [39]. In this study, recent advances in probe specificity and detection sensitivity allowed the correlation of connexin mRNA and protein levels with breast cancer prognosis at unique quality. We disclosed the expression of five connexin isotypes by confirming the production of GJA1/Cx43, GJA3/Cx46 and GJB2/Cx26 [27], [31], [40]–[42] and detecting, for the first time, GJA6/Cx30 and GJB1/Cx32 both in the human pre-menopausal mammary gland and breast carcinomas. Transcriptomic analysis of both array datasets, Affymetrix and Illumina of ∼2000 patients each, cross-validated each other’s results. In line with the correlations found at mRNA level, elevated Cx43 protein levels were linked with significantly improved breast cancer outcome, offering Cx43 protein detection as an independent prognostic marker outperforming vascular invasion or necrosis. As a contrary, elevated Cx30 mRNA and protein levels were associated with a reduced disease outcome offering Cx30 protein detection as an independent prognostic marker outperforming MI or necrosis. The prognostic value of the rest of connexin isotypes revealed at mRNA level was either lost (Cx46) or was discordant (Cx26 and Cx32) with that observed at protein level suggesting a complex regulation of these isotypes involving significant post-transciptional/−translational control for further clarification. These data show that differential connexin expression may serve as a potential marker of breast cancer prognosis.

Most data on the regulation of connexin expression are related to Cx43 [43], which had good prognostic correlations between mRNA and protein levels in this study. The tested gene array data suggest that in ER positive primary breast cancers Cx43 mRNA expression can be linked with tumor suppression [44], while in ER negative cases with tumor protection [33]. 17β estradiol through ERα can promote both the proliferation of mammary epithelial cells [45] and the expression and functions of Cx43 channels, which are implicated in cell cycle control [46], [47]. The potential control of tumor growth by Cx43 is likely to contribute to the better differentiation and improved patient survival of ER positive tumors [48]. In ER negative breast cancers other pathways dominate in Cx43 expression potentially involving Wnt-1 and/or Ras-Raf-MAPK activation [43], which can also be part of mitogenic signaling responsible for the less differentiated phenotype and worse prognosis [49]. In these advanced tumors, connexins can also contribute to metastatic invasion, transendothelial diapedesis and colonization of breast cancer cells in line with the observations made by several studies [19], [32], [33], [50], [51]. ER subtypes may also influence disease outcome [52] since activating ERβ may suppress both Cx43 expression and tumor growth [46]; so as posttranslational regulation since Cx43 protein levels were not linked to a prognostic inversion seen at mRNA level in ER negative cases. The prognostic value of Cx43 was preserved after hormone therapy, implying that Tamoxifen or aromatase inhibitors can block mitogenic signaling [49] without significantly reducing Cx43 expression and functions.

Elevated Cx30 transcript levels showed almost complete inverse prognostic correlations compared to those of Cx43 with a reduced survival in ER positive tumors and a strong trend for better outcome in HER2 and triple negative cancers, consistent with a complementary regulation of these connexins in breast carcinogenesis and progression. Cx46 mRNA expression was also linked with improved survival in ER positive patients which, however, was preserved also in ER negative patients, suggesting a reciprocal regulation of Cx46 compared to Cx43 expression in this subgroup. This is in line with data gained in lens cell cultures showing the simultaneous down-regulation of Cx43 and upregulation of Cx46 expression by the tumor promoter phorbol ester (12-O-tetradecanoylphorbol-13-acetate) [53] or by the activation of the MAPK/ERK pathway [54]. Differential regulation of connexin isotypes are rather common and allows connexins to serve either as conditional tumor suppressors in primary cancer or tumor supporters in advanced, metastatic cases [30], [32], [33], [51]. Examples in gynecological tumors include the re-expression of Cx26 induced by phorbol ester and its down-regulation via PR, which induce the opposite effects on Cx43 [40], [55], [56]. Also, 17β estradiol promotes Cx43 expression and function [46] but reduces Cx26 and Cx32 production and activities [57].

The cytoplasmic connexin protein we detected in breast cancers can be compatible with the potential channel independent functions of connexins observed in malignant tumors [14], [50]. Tight correlations were found between mRNA and protein expression and relatively coherent links between these and breast cancer prognosis for connexin subtypes which were either localized to the myoepithelial layer only (Cx43) or both to myoepithelial and luminal epithelial cells (Cx46 and Cx30) in normal mammary glands, as opposed to the luminal epithelia related Cx26 and Cx32, which showed partly discordant prognostic links. This suggests that connexin expression in myoepithelia is more consistently regulated than in luminal cells at least in a malignant tumor setting. Accordingly, in line with mRNA data, elevated Cx43 and Cx30 protein levels were linked with better and poorer RFS, respectively, in the whole patient cohort. Most significantly, differential connexin expression allowed the prognostic stratification of grade 2 patients into good or poor RFS subgroups by *is situ* testing of Cx43 levels; or grade 3 patients into poor or good OS subgroups by monitoring Cx30 levels. Potential prognostic relevance of connexins was indirectly supported by the positive statistical link of Cx43 protein levels with ER positivity and its negative link with tumor grade and by the positive link between Cx30 protein levels and mitotic index. As a contrary, Cx26 linked with worse outcome in luminal B patients at the transcript level, was associated with better RFS in the whole cohort and in ER positive patents at the protein level. Also, though Cx32 expression was linked with improved RFS at the transcript level, it showed an inverse prognostic link at the protein level in most breast cancer subgroups.

Because of the close correlations between the results of independent mRNA array platforms and the validated specificity of the connexin antibodies used by western blots, the discordant prognostic links between mRNA and protein levels may not be associated with defective probes or techniques. Besides transcription factors and epigenetic processes, post-transcriptional pathways can also be involved in the regulation of connexins in breast cancer, which may more significantly affect Cx26 and Cx32 than other isotypes [58]. These include micro-RNAs, since there are multiple micro-RNA binding sites in the 3′-untranslated regions of connexin genes for cell-type-specific regulation of connexin protein levels [59]. Tumor stage dependent differential degradation including lysosomal, autophagy mediated or proteasomal mechanisms, such as described by the interaction of Cx43 and TRIM21 an E3 ubiquitin-protein ligase, can modify connexin levels [60], [61], which need further clarification in tumor development and progression. Furthermore, these and further posttranslational modifications including phosphorylation, SUMOylation, nitrosylation, hydroxylation, acetylation or methylation of connexins, may alter protein conformation, which can diversely affect recognition of antigenic epitopes by the antibodies we used [62], [63]. We previously showed that reduced Cx26 protein levels were linked with improved prognosis after neoadjuvant chemotherapy where Cx32 protein detection had no prognostic impact [7]. Other studies came to the opposite conclusion by linking the loss of Cx26 expression to reduced survival in primary gastric and colorectal carcinomas [64], [65]. Therefore, Cx26 and Cx32 may not be stable markers and their prognostic relevance in cancer should be interpreted with particular care by considering tumor type, stage and treatment.

The cell membrane association of connexins, we detected in the normal mammary glands, is compatible with functioning channels, which can be formed only between connexins of the same subfamilies, either within GJA or GJB classes (***see*** ***Fig. 3***) [66]. Based on our findings, compatible connexins detected in the cell borders, which likely involve the cell membranes, can potentially form heterocellular channels in the normal mammary gland to be further clarified. Theoretically, myoepithelial Cx43 (GJA1) can form heterocellular channels with Cx46 (GJA6) but not with Cx30 (GJB6) of the myo- and luminal epithelium. Cx26 (GJB2) and Cx32 (GJB3) can also form homo- and heterotypic/heretomeric channels between luminal cells [67] and heterocellular channels with the myoepithelial Cx30 [68], [69]. This complexity and the differential regulation of connexins offer a substantial plasticity for the fine regulation mammary gland functions including cyclic proliferation, regression and lactation, through connexin channels [28].

In conclusion, the selective expression and compatibility of the five connexin isotypes revealed in human mammary epithelial layers allow complex regulation of glandular functions through direct cell-cell communication. In breast cancer, the differential expression of connexins either at mRNA and protein level may be used for the potential prognostic stratication of tumor subtypes. In particular, Cx43 and Cx30, which respectively show positive and negative prognostic values concordant between mRNA and protein levels, offer themselves as potential markers of breast cancer outcome.

## Supporting Information

Figure S1 **Separation of patients into high and low mRNA expression groups along the median connexin expression for testing correlations between connexin transcript levels and breast cancer prognosis.** Data collected from Affymetrix platform (a) and from the Illumina platform (b).
(TIF)
