# Supplementary figures and images for: Correlations of Differentially Expressed Gap Junction Connexins Cx26, Cx30, Cx32, Cx43 and Cx46 with Breast Cancer Progression and Prognosis
Source: PLoS One. 2014 Nov 10;9(11):e112541. doi: 10.1371/journal.pone.0112541 (PMC4226536; doi:10.1371/journal.pone.0112541)

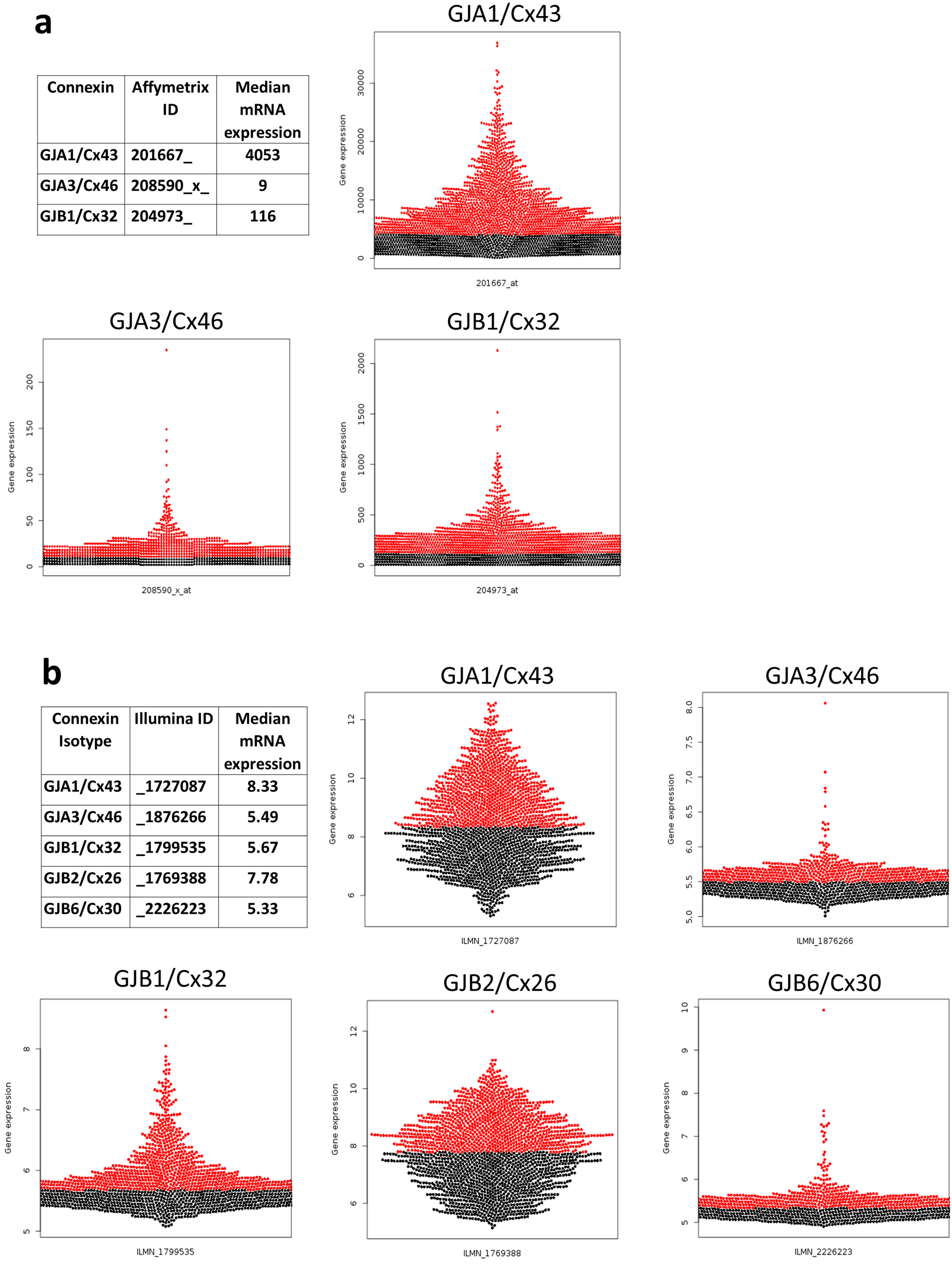

Supplement: Figure S1 — Separation of patients into high and low mRNA expression groups along the median connexin expression for testing correlations between connexin transcript levels and breast cancer prognosis. Data collected from Affymetrix platform (a) and from the Illumina platform (b). (TIF) [file pone.0112541.s001.tif]
